# Investigating the impact of TB case-detection strategies and the consequences of false positive diagnosis through mathematical modelling

**DOI:** 10.1186/s12879-018-3239-x

**Published:** 2018-07-21

**Authors:** Marek Lalli, Matthew Hamilton, Carel Pretorius, Debora Pedrazzoli, Richard G. White, Rein M. G. J. Houben

**Affiliations:** 1 Department of Infectious Disease Epidemiology, Keppel Street, WC1E 7HT, London, UK; 2 Avenir Health, Glastonbury, USA

**Keywords:** Tuberculosis, Screening, False positive diagnosis, Mathematical modelling

## Abstract

**Background:**

Increasing case notifications is one of the top programmatic priorities of National TB Control Programmes (NTPs). To find more cases, NTPs often need to consider expanding TB case-detection activities to populations with increasingly low prevalence of disease. Together with low-specificity diagnostic algorithms, these strategies can lead to an increasingly high number of false positive diagnoses, which has important adverse consequences.

**Methods:**

We apply TIME, a widely-used country-level model, to quantify the expected impact of different case-finding strategies under two scenarios. In the first scenario, we compare the impact of implementing two different diagnostic algorithms (higher sensitivity only versus higher sensitivity and specificity) to reach programmatic screening targets. In the second scenario, we examine the impact of expanding coverage to a population with a lower prevalence of disease. Finally, we explore the implications of modelling without taking into consideration the screening of healthy individuals. Outcomes considered were changes in notifications, the ratio of additional false positive to true positive diagnoses, the positive predictive value (PPV), and incidence.

**Results:**

In scenario 1, algorithm A of prolonged cough and GeneXpert yielded fewer additional notifications compared to algorithm B of any symptom and smear microscopy (*n* = 4.0 K vs 13.8 K), relative to baseline between 2017 and 2025. However, algorithm A resulted in an increase in PPV, averting 2.4 K false positive notifications thus resulting in a more efficient impact on incidence. Scenario 2 demonstrated an absolute decrease of 11% in the PPV as intensified case finding activities expanded into low-prevalence populations without improving diagnostic accuracy, yielding an additional 23 K false positive diagnoses for an additional 1.3 K true positive diagnoses between 2017 and 2025. Modelling the second scenario without taking into account screening amongst healthy individuals overestimated the impact on cases averted by a factor of 6.

**Conclusion:**

Our findings show that total notifications can be a misleading indicator for TB programme performance, and should be interpreted carefully. When evaluating potential case-finding strategies, NTPs should consider the specificity of diagnostic algorithms and the risk of increasing false-positive diagnoses. Similarly, modelling the impact of case-finding strategies without taking into account potential adverse consequences can overestimate impact and lead to poor strategic decision-making.

**Electronic supplementary material:**

The online version of this article (10.1186/s12879-018-3239-x) contains supplementary material, which is available to authorized users.

## Background

The End TB Strategy calls for a 95% reduction in deaths due to tuberculosis (TB) and a 90% reduction in incidence rate by 2035, relative to 2015 [1]. However, the annual rate of decline of global incidence is estimated at 1.4% per year between 2000 and 2016 and must be accelerated to 10% to reach the 2025 milestone and then to 17% to reach the 2035 target of the End TB Strategy [2, 3]. Individuals with active TB disease that experience diagnostic delays or remain undetected not only fail to access the care and treatment they need, but also contribute to continued transmission, which hinders progress toward the global targets.

Since implementation of the Directly Observed Therapy Short-course (DOTS) Strategy in the 1990s, a strong focus has been placed on case notification, which continues to be one of the primary indicator of programme performance [4]. Recognising the need to detect more cases, NTPs in low- and middle-income countries often need to target screening efforts toward populations at increasingly lower prevalence of disease. There has been a push to complement passive case finding with more active community-based approaches, in an attempt to reduce transmission by finding and treating cases faster [5, 6]. However, the current body of evidence demonstrating a population-level epidemiological impact attributable to community-based active case finding (ACF) is extremely limited [7–9].

The pressure to detect more cases in order to reach ambitious targets may cause oversight of the positive predictive value (PPV) of the cases detected. A low prevalence of TB disease in target populations results in a high number needed to screen (NNS) in order to find one case. This can lead to high screening costs and concerns of increasing false positive diagnoses as prevalence falls, given the imperfect specificity of diagnostic algorithms. In order to limit these negative effects, the World Health Organization (WHO) recommends taking a targeted approach to ACF by considering the prevalence of disease in the screening population, using diagnostic algorithms with higher accuracy, and carefully assessing the potential NNS and resulting PPV of the detected population [10].

Given the range of possible combinations of screening and diagnostic tools as well as target populations, National TB Control Programmes (NTPs) face difficult decisions in allocating limited resources toward screening strategies that will maximise impact. Furthermore, programmes are challenged to improve their diagnostic algorithms, needing to carefully assess the potential trade-off between net sensitivity and specificity. These decisions are often based on epidemiological principles using data from diagnostic accuracy studies, without quantifying the potential impact of different strategies. Mathematical modelling can provide impact estimates for different policy options and contribute to evidence-informed policymaking, but few models are designed for use by domestic programme planners without formal training in modelling. Of the models available, most lack parameters to allow for screening amongst individuals without TB disease and the specificity of the diagnostic algorithm. These parameters are key structural components for a model to account for false positive diagnoses – a critical consideration when evaluating case-finding strategies.

One available model is the ‘ScreenTB’ tool, developed by WHO. ScreenTB is available as an online platform to estimate the potential yield of different systematic screening scenarios, based on the diagnostic algorithm and risk of TB in the target population [11]. Screen TB is a static model, and therefore cannot account for dynamical changes in yield following the first round of screening, or changes in the screening population’s composition over time. The TB Impact Model and Estimates (TIME) is a user-friendly country-level tool designed to be used by domestic programme planners for strategic planning of TB care and prevention activities [12]. TIME is a dynamic transmission model with screening structure that allows programme planners to investigate how notifications, PPV, and impact on burden estimates may change over time with the introduction of different case-finding approaches or diagnostic algorithms. Additionally, programme planners can visualise the care cascade under different scenarios to evaluate numbers screened, tested, diagnosed, linked to care, and successfully treated, in order to identify possible gaps in programmatic activities.

In this study, we use TIME to assess the potential impact of two different scenarios, and demonstrate the importance of considering the potential for false positive diagnoses in both TB programme planning and mathematical modelling. The examples presented here are based on real-life scenarios the authors experienced when providing technical assistance with TIME to TB programmes during the national strategic planning process. The data and scenarios were adapted for anonymity and clarity.

## Methods

### The TIME model

Technical aspects of the TIME model and its structure are previously described elsewhere [12]. In summary, TIME Impact is a deterministic, compartmental TB transmission model. The core model contains a compartment for susceptible individuals, latent infection and two compartments for active disease, one for smear positive disease and one for smear negative disease. Movement of individuals between compartments are governed by parameters which reflect the natural history of TB. The model is stratified by HIV status (HIV negative vs HIV positive, on or off anti-retroviral therapy), treatment history (new vs retreatment) and drug resistance status (drug-susceptible vs multi-drug resistance). The demographic component is informed by UN Population Division estimates and is stratified by sex and age by five-year age bins, with separate TB natural history parameters for under-15 year olds to account for the different epidemiological properties of paediatric TB.

### Model calibration

We used an existing model calibrated to a West African setting of low HIV prevalence. The model is calibrated to notifications, prevalence, incidence and mortality. Programmatic data used to parameterise the model were collated in collaboration with the NTP both remotely and during several in-country visits. Estimates of prevalence were taken from the recent national prevalence survey, whereas estimates of incidence and mortality were extracted from the WHO Global TB Database [13].

**Fig. 1:**
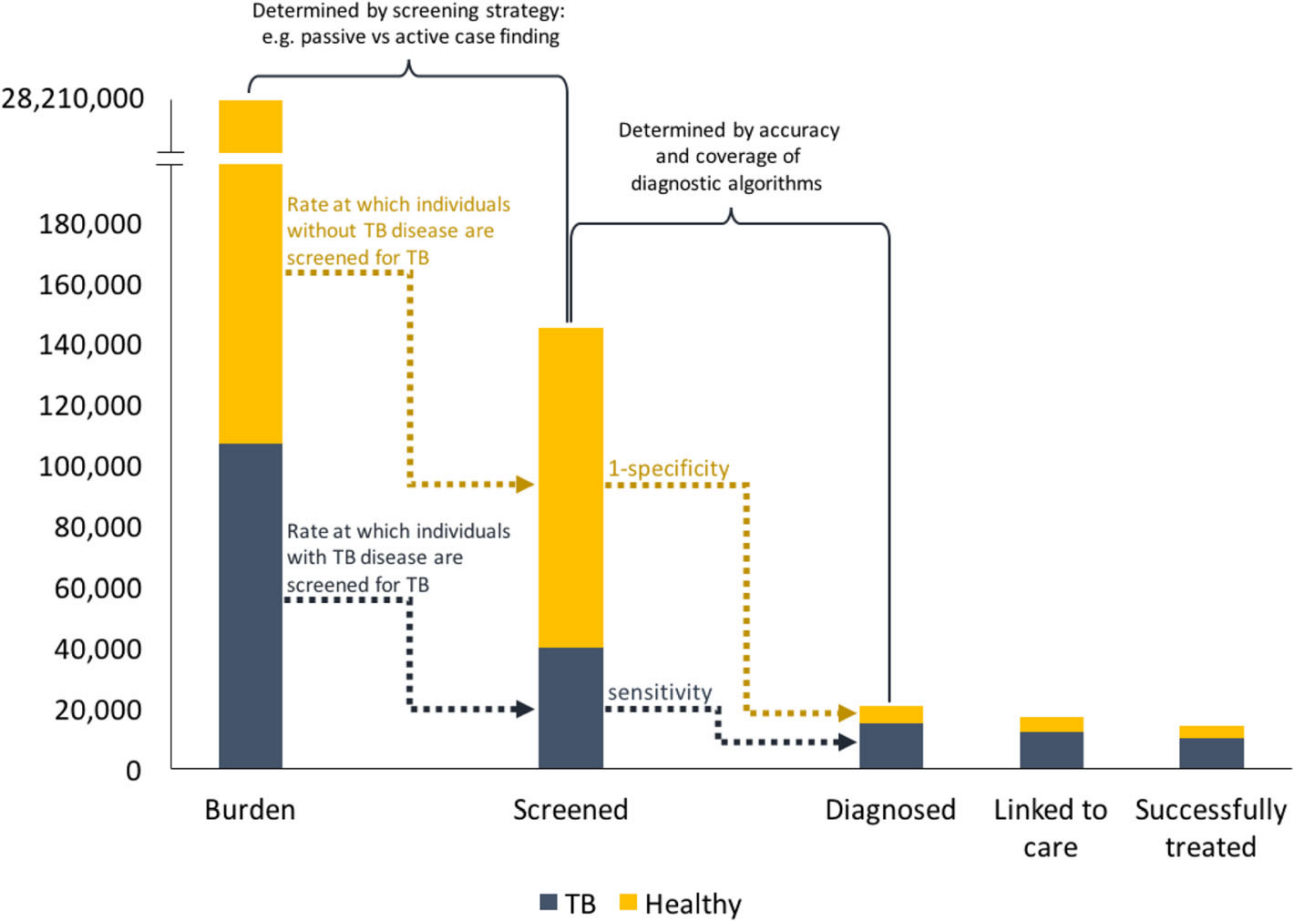
Care cascade for baseline projection in 2017

The calibrated model represents the status quo if TB programmatic activities and investments remain unchanged from the baseline year in 2017. The baseline diagnostic algorithm that is implemented nationwide in this setting is prolonged cough (≥ 2 weeks) followed by smear microscopy, and clinical diagnosis for smear-negative test results. The estimated net sensitivity and specificity of the algorithm are found in Table 1. We estimate outcomes between 2017 and 2025 to serve as baseline for comparison with modelled case-finding intervention scenarios. A visualisation of the care cascade in the baseline projection for 2017 is shown in Fig. 1, illustrating the composition of healthy and disease individuals at each step of the cascade. The first three bars of the care cascade relate to case finding. Programmatic screening efforts and target population drive the change in composition between the ‘Burden’ and ‘Screened’ bars. The sensitivity of the diagnostic algorithm drives the attrition of TB diseased individuals (grey component) between the ‘Screened’ and ‘Diagnosed’ bars, whereas the specificity of the diagnostic algorithm filters out the healthy individuals (yellow component).

**Table 1:** Net sensitivity and specificity of diagnostic algorithms reflected in each scenario, by smear type. Green cells represent large increase in value compared to baseline algorithm

Scenario		Definition	Net sensitivity		Net specificity
			Smear positive	Smear negative	
Baseline		Prolonged cough & microscopy/clinical diagnosis	50.0%	20.9%	94.9%
Scenario 1	Algorithm A	Prolonged cough & GeneXpert	49.1%	27.8%*	99.9%*
	Algorithm B	Any symptom & microscopy/clinical diagnosis	77.0%*	20.9%	94.3%
Scenario 2	Microscopy (baseline)	Prolonged cough & microscopy/clinical diagnosis	50.0%	20.9%	94.9%
	GeneXpert	Prolonged cough & GeneXpert	49.1%	27.8%*	99.9%*

*Asterisk signifies large increase in value compared to baseline algorithm; Scenario 1 comparing the impact of two different diagnostic algorithms in a defined population; Scenario 2 examining the impact of expanding case detection towards population of lower disease

Data informing the sensitivity and specificity of symptoms screening were obtained from a previously-published meta-analysis and are consistent with those used by WHO as recommendations for systematic screening of active TB and for the ScreenTB tool [10, 14]. Estimates of sensitivity and specificity of diagnostic components of the algorithms were obtained from various sources and are consistent with WHO recommendations [10, 15, 16].

In both intervention scenarios, we assumed linear scale-up of activities between 2017 and the target year of 2020, and assumed constant values from 2020 onward. We recorded absolute number of additional notifications, positive predictive value, and population-level impact on TB incidence between 2017 and 2025 (the second milestone of the End TB Strategy targets). We used these data to create two metrics of efficiency for comparison between scenarios: 1) the ratio of additional true positive to additional false positive notifications, which describes how many false positive diagnoses are notified (or averted) for every additional true positive diagnosis, relative to baseline; 2) the ratio of total notifications to cases averted, which describes how many cases are averted, relative to baseline, per case notified.

### Scenario 1: Comparison of two diagnostic algorithms

Scenario 1 represents a comparison of implementing two different diagnostic algorithms in a population with similar prevalence of TB disease and access to care, with a pre-test probability of 28%. In this scenario, the country plans to increase coverage of case-finding activities in order to increase the total population tested by 20%, using either Algorithm A of prolonged cough (≥ 2 weeks) followed by GeneXpert, or Algorithm B of any symptom followed by smear microscopy and clinical judgement in the case of smear negative results. This scenario investigates the impact of increasing the net sensitivity alone (Algorithm B) versus increasing both the net sensitivity and specificity (Algorithm A).

### Scenario 1 parameterisation

The two diagnostic algorithms under investigation and their calculated net sensitivities and specificities for smear positive and smear negative TB are found in Table 1. The rate at which individuals with active TB disease are screened for TB is increased in the target year until the number individuals tested for TB was 120% that of the baseline value in 2017. The pre-test probability is assumed to be constant. Inputs for the average net sensitivity and specificity are calculated based on the population coverage of the algorithms. See Additional file 1 for model parameters.

### Scenario 2: Expand intensified case-finding activities nationwide

Scenario 2 represents a projection of impact from expanding intensified case-finding (ICF) activities in the outpatient department (OPD) nationwide, based on data from a pilot study in a high-prevalence urban setting. In this scenario, the country aims to investigate the potential impact of reaching their notification targets using a defined and tested case-finding strategy. The country makes use of data from a pilot study, demonstrating that an additional 50% of individuals were considered presumptive TB and were tested for disease, which increased notifications by 20% in the pilot site. The programme would like to use modelling to investigate the potential total notifications and epidemiological impact if this strategy is implemented nationwide. Other data sources include surveillance data stratified by administrative division, and results from a recent national prevalence survey with urban-rural stratification.

### Scenario 2 parameterisation

We assume that the pre-test probability amongst individuals screened through ICF activities in the pilot study is 2%, based on operational research in South Africa and the country’s prevalence of disease in the general population [17]. To reflect the change of population away from the high-prevalence urban setting, we assume that the prevalence of disease and pre-test probability are half those of the test site, informed by prevalence survey findings. Routine surveillance data from the country suggests that 70% of diagnostic tests for TB are reported in urban areas, which is used to stratify the parameterisation by urban and rural setting. We assume that the pilot data reflects the average of urban diagnostic centres and a similar 50% increase in the number of individuals tested for TB across implementing sites. Table 1 shows the sensitivity and specificity of the diagnostic algorithms, which are kept constant over time. See Additional file 1 for parameter details.

We investigated the potential impact of this intervention if smear microscopy was replaced by GeneXpert. In this scenario, we assume the same screening rate as in the microscopy scenario, but increase the sensitivity and specificity of the algorithm to reflect an ICF scenario where GeneXpert is being scaled up as primary diagnostic test, replacing smear microscopy by 2020.

Finally, we repeated the scenario of expanding ICF activities with smear microscopy to reflect the impact of modelling case-finding interventions using a model that does not have structure to allow for false positive diagnoses, i.e. specific model parameters related to screening amongst individuals who do not have TB disease as well has the specificity of diagnostic algorithms. Here we modelled a scenario where the increase in notifications from the pilot study are all true positive, and disregard screening amongst individuals without disease. We investigated the epidemiological impact by increasing the screening rate to match a 20% increase in notifications in a model that does not account for the specificity of the algorithm, therefore assuming that all additional notifications are from the diseased population only.

## Results

### Scenario 1

In this scenario, we investigated the epidemiological impact of two different diagnostic algorithms, where Algorithm A represents prolonged cough (≥2 weeks), followed by GeneXpert and Algorithm B represents screening for any symptom, followed by sputum smear microscopy or clinical diagnosis for smear negative patients.

**Fig. 2:**
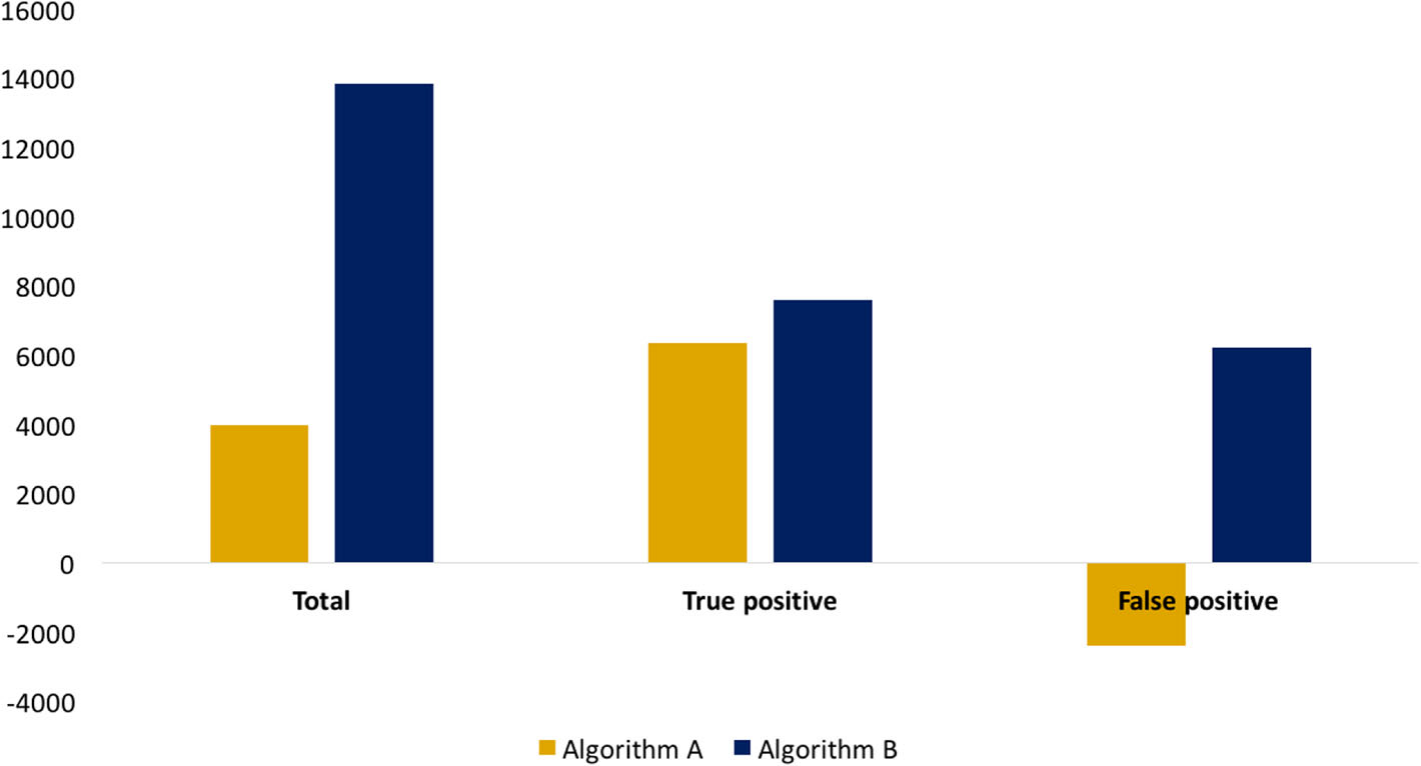
Additional notifications between 2017 and 2025 based on diagnostic algorithm

Both algorithms result in an increase in total notifications relative to baseline; however, between 2017 and 2025, the increase from Algorithm B is 3.5 that of Algorithm A (Fig. 2, yellow vs. blue bars). Similarly, Algorithm B has a greater impact on incidence, averting 64% more cases than Algorithm A between 2017 and 2025. Projections of total notification rate, incidence rate, number of false positive notifications and PPV can be found in Additional file 1.

The larger reduction on incidence by Algorithm B can be explained by the higher net sensitivity of the algorithm, which yields a greater increase in true positive notifications than Algorithm A, relative to baseline (Fig. 2). However, the increased net specificity of Algorithm A compared to the baseline algorithm (Table 1) results in a prevention of false notifications, with a ratio of − 0.4 false positive notification for every additional true positive notification, i.e. for every additional true positive notification, 0.4 false positive notifications were averted. On the other hand, Algorithm B results in further false positive notifications per additional true positive notification with a ratio of 0.81 to 1, i.e. for every additional true positive notification, there are 0.81 additional false positive notifications, relative to baseline.

The algorithms’ difference in specificity leads to diverging impacts on PPV. During 2017–2020, Algorithm A increases the PPV from 74 to 77%, while Algorithm B results in PPV decline from 74 to 73%. Beyond 2020, the PPV of both algorithms decline, although Algorithm A maintains a higher PPV in 2025 than both baseline and Algorithm B. Algorithm A has a more efficient impact on incidence with 0.27 additional notifications needed to avert one case, as compared with 0.57 additional notifications under Algorithm B (Table 1).

### Scenario 2

In this scenario, we investigated the impact of expanding intensified case-finding activities nation-wide from a high-prevalence urban area (ICF with microscopy) as well as the impact of replacing sputum smear microscopy by GeneXpert (ICF with GeneXpert).

**Fig. 3:**
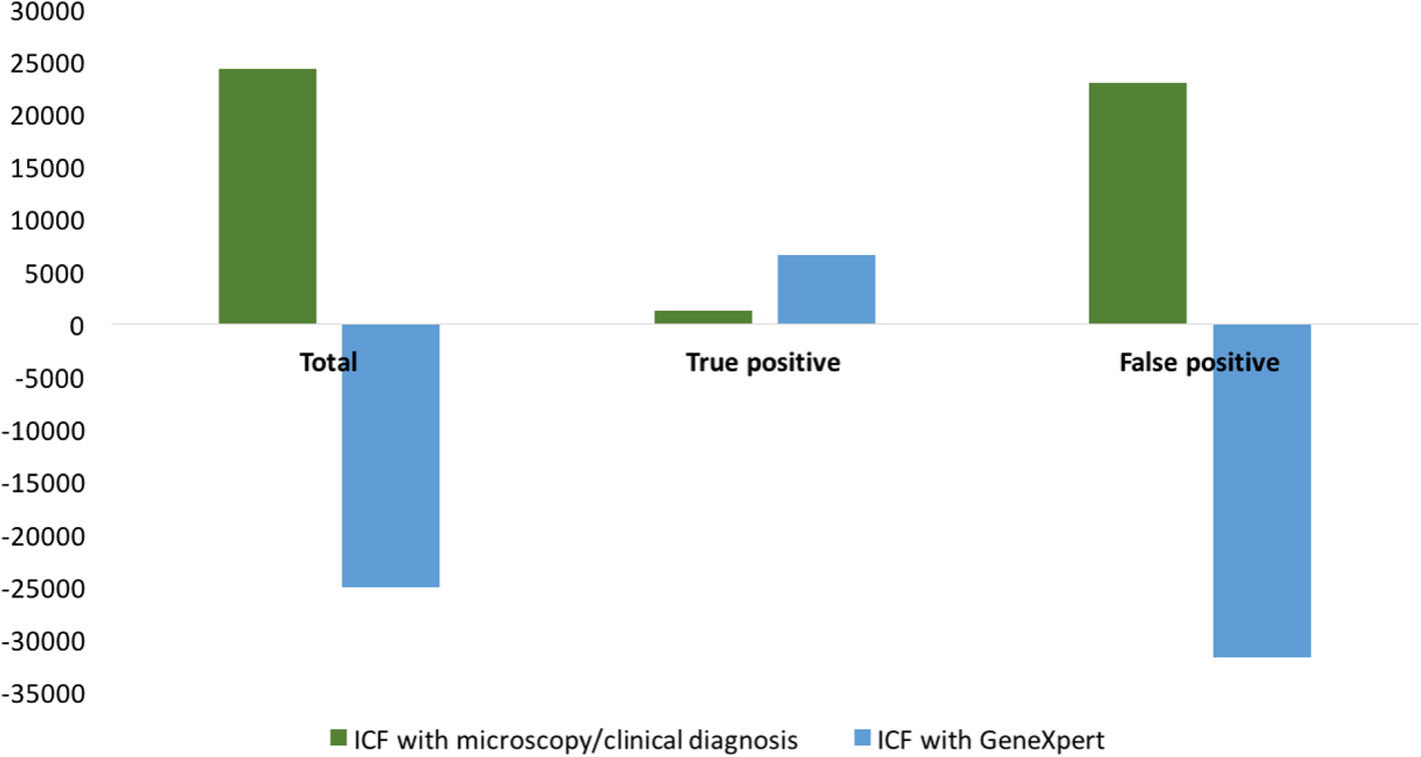
Additional notifications between 2017 and 2025 of ICF activities based on diagnostic test

Projections for total notification rate, incidence rate, number of false positive notifications and PPV can be found in Additional file 1. Expanding ICF activities nationwide yields an additional 24.3 K notifications between 2017 and 2025, relative to the baseline scenario, if microscopy is used as the final diagnostic test (green bar, Fig. 3). However, the expansion with microscopy averts only 3.6 K cases over the period, and leads to a high FP:TP ratio of 17.7 additional false positive notifications per additional true positive notification.

With GeneXpert as the final diagnostic test, the expansion averts 5.0 K cases relative to baseline over the period, with a lower FP:TP ratio of − 4.8 additional false positive notifications per additional true positive notification relative to baseline, suggesting that ICF with GeneXpert leads to a prevention of false positive notifications relative to the status quo. The result is a large increase in the PPV to 99% by 2020 with GeneXpert, compared to a decrease in PPV to 63% in 2020 with smear microscopy (baseline PPV = 74% in 2020). In other words, 37% of the total notifications could be false positive if microscopy is used in this scenario. Therefore, the model shows that including GeneXpert in the ICF scenario leads to a more efficient impact on incidence, by increasing true positive notifications and decreasing false positive notifications for a net decrease in total notifications (Fig. 3, blue bars); while still having a larger impact on incidence compared to if microscopy was used (Table 2).

**Table 2:** Modelled outcomes between 2017 and 2025 for each scenario

Scenario		Additional notifications between 2017 and 2025 (absolute numbers, × 1000)			Number of cases averted between 2017 and 2025 (× 1000)	Percent reduction in incidence by 2025 compared to 2017	Absolute change in PPV by 2025 compared to baseline	Number of additional FP notifications per additional TP notification	Number of additional notifications needed to avert one case
		Total	TP	FP					
Scenario 1	Algorithm A	4.0	6.4	−2.4	14.7	8.4%	+ 2	−0.4	0.3
	Algorithm B	13.8	7.6	6.2	24.2	12.2%	−5	0.8	0.6
Scenario 2	ICF with microscopy	24.3	1.3	23.0	3.6	3.6%	−11	17.7	6.7
	ICF with GeneXpert	−25.1	6.6	− 31.7	5.0	4.3%	+ 36	−4.8	−5.0

*TP* True positive notification; *FP* False positive notification; *PPV* Positive predictive value; Scenario 1 comparing the impact of two different diagnostic algorithms in a defined population; *Algorithm A* Prolonged cough & GeneXpert; *Algorithm B* Any symptom & microscopy/clinical diagnosis; *Scenario 2* Examining the impact of expanding case detection towards population of lower disease

**Fig. 4:**
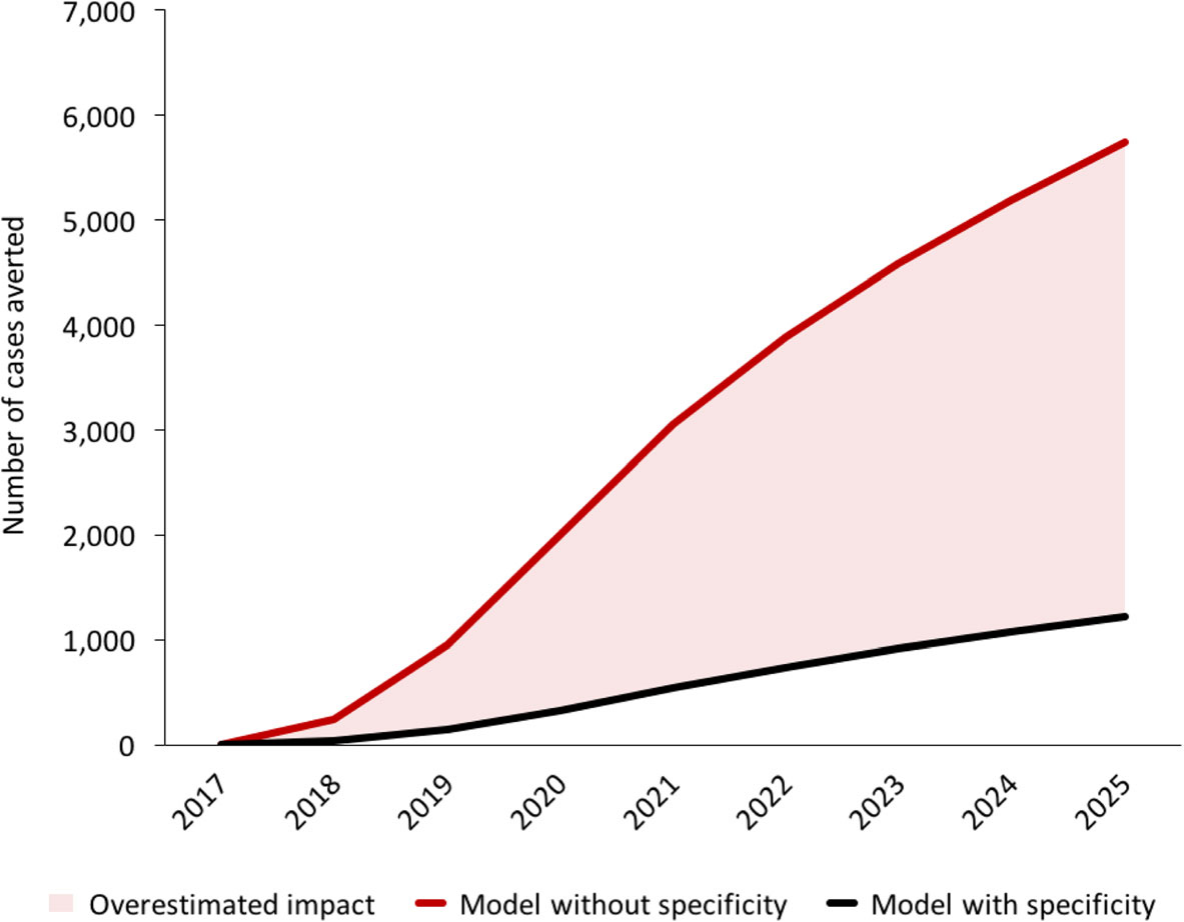
Modelled number of cases averted, with and without considering screening amongst healthy individuals

Modelling the ICF with microscopy scenario without taking into consideration the possibility of false positive diagnoses overestimates the population-level impact on cases averted by a factor of 6 between 2017 and 2025. In Fig. 4, the pink shaded region depicts the overestimated impact resulting from the use of a model that does not take specificity into account. Such a model would necessarily assume that the entire 20% increase in notifications is drawn from the TB-diseased population, without taking into account the 50% increase in people tested for TB or the split in pre-test probability. As a result, the model would remove too many infectious individuals from the prevalent pool and dramatically overestimate the reduction in transmission.

## Discussion

This modelling study demonstrates how the net accuracy of diagnostic algorithms and the prevalence of the target population can influence notifications and epidemiological impact, via changes in PPV. This is consistent with findings from other modelling studies [18, 19].

These examples demonstrate the adverse consequences of neglecting the PPV. False positive diagnoses become unnecessarily treated non-TB diseased individuals, wasting health system resources and harming those individuals by exposing them to toxic drugs, possible stigma, and possible catastrophic costs related to accessing and adhering to TB care [20–23]. Depending on the sensitivity of the definition across different settings and prevalence of associated risk factors, hepatotoxicity due to anti-tuberculosis chemotherapy has been reported in between 2 and 28% of TB cases [24]. In resource constrained settings, which carry the highest burden of TB, monitoring of liver enzyme levels throughout TB treatment is not routinely done. This means that individuals in these settings are at risk of not receiving the appropriate and timely management of hepatotoxicity, which, if left unmanaged, can lead to a reduced effectiveness of anti-tuberculosis treatment and an increase in morbidity and mortality [24, 25].

False positive diagnoses may also create the illusion of progress toward programmatic goals related to case detection that are set in national monitoring and evaluation frameworks and reported to the global level. Furthermore, national and global figures for treatment outcomes may be biased towards favourable outcomes if individuals without TB disease are more likely to be reported as having a successful treatment for a disease they never had. While we have not carried out a formal cost-effectiveness analysis, it is reasonable to assume that false-positive diagnoses reduce the cost-effectiveness of interventions.

Total number of notifications is widely used as an indicator of programme performance for TB programmes globally. However, considering total number of notifications alone, without taking into account the PPV, could mislead programme planners and result in suboptimal planning for TB case-finding. Rather than focussing exclusively on increasing total notifications, TB programmes should aim to maximize their impact on TB burden. Specifically, more consideration can be given to other monitoring indicators, such as the proportion of bacteriological confirmation, when assessing programme performance. For example, a higher coverage of bacteriological confirmation would imply a larger impact on burden, due to the lower risk of false positivity and thus a higher PPV. As demonstrated by the modelled scenarios, greater yield in total notifications does not necessarily translate into greater epidemiological impact on disease burden. Algorithm A in Scenario 1 yielded fewer additional notifications than did Algorithm B, but improved the PPV by avoiding false positive diagnoses. As a result, the impact on burden of Algorithm A was achieved with much greater efficiency.

When mathematical models are used to inform strategic planning related to case-detection, it is critical that they include the structure to distinguish false positive notifications. The TB Modelling and Analysis Consortium (TB-MAC) recently developed a set of guiding principles for TB country-level modelling and its use to support the policy process [26]. One of the principles is appropriateness of model structure – which states that models should be appropriately designed to represent key features for capturing TB dynamics and modelling the impact of the interventions at question. This guiding principle is not respected if a model is unable to distinguish false positive notifications, and therefore should not be used for the intent of guiding policy decisions for case detection.

In their efforts to find more cases, TB programmes are often forced to expand case-finding strategies to target populations at low prevalence of disease. In Scenario 2, the TB programme initiated ICF in the outpatient department in an urban setting, and used these data to model the expansion of the activities to rural settings with a lower prevalence of disease. In a situation like this, diagnostic specificity becomes critical. The use of microscopy and clinical diagnosis resulted in 37% false positivity during 2017–2020, as compared with 1% false positivity using GeneXpert. The large reduction in false positive diagnoses through GeneXpert, translated to a reduction in total notifications compared to baseline. This finding is consistent with other studies, which showed that implementation of GeneXpert may not necessarily lead to an increase in total notifications [27, 28]. The use of GeneXpert in our example had only a marginally larger impact on incidence compared to microscopy. This can be explained by the low sensitivity of the initial screening test of prolonged cough, which severely limits the sensitivity and therefore the entire algorithm’s potential to find cases. If a more sensitive initial screen was used, such as any symptom consistent with TB, then the epidemiological impact would have been greater, as more individuals with TB disease would have been found.

We assumed that the impact achieved through the pilot study is reflective of all urban settings. However, it’s important to consider the potential loss of impact when expanding outside of pilot study conditions and into programmatic implementation; therefore, our example may be overestimating the outcomes.

The current model does not take into account uncertainty in natural history parameters. While uncertainty may impact specific output values, the direction of the modelled impact would hold and the overall messages in this paper would remain the same.

Modelling the impact of case-finding strategies is limited by the data available, such as the quality of sensitivity and specificity data, estimates of disease prevalence in various target populations and how individuals contact each other within and between different populations.

The process and accuracy of diagnosing TB based on clinical evaluation are not well understood. However, clinical diagnosis continues to play an important role in TB case detection, given existing barriers (e.g. distance) to accessing laboratory-based diagnostic services, as well as difficulties in collecting specimen in certain populations (e.g. paediatric). Clinical diagnostic practices can differ between countries as well as between individuals. This variability makes it difficult to draw generalisable conclusions on its accuracy. Furthermore, the accuracy of clinical diagnosis, and the decision to treat empirically, is influenced by the use and interpretation of chest radiography, which can increase the sensitivity of clinical diagnosis at the expense of specificity, potentially leading to more false positive diagnoses [16]. However, quantifying the relationship remains a challenge. Modelled outputs are very sensitive to changes in the diagnostic accuracy parameters, particularly to changes in specificity, as this relates to individuals without disease who constitute the majority of individuals screened. Programme planners can use TIME to explore the impact of different accuracy parameter values within the bounds of available estimates to reflect different clinical practices regarding empirical treatment. Further research is needed to gain a better understanding of accuracy of the tools recommended by WHO, and especially of clinical diagnosis, which is widely used and a potentially important source of false positivity. The accuracy estimates referred to in WHO guidance documents and ScreenTB tool can serve as a resource for TB strategic planning; however, programme planners should make use of setting-specific estimates based on local data, if appropriate.

## Conclusion

This modelling study demonstrates the importance of taking into account possible false positive diagnoses when choosing a case-finding strategy and when interpreting changes in total notifications as indicator for programme performance. NTPs must carefully consider the specificity of diagnostic algorithms before expanding case-finding activities to populations with lower prevalence of disease. Furthermore, failing to consider the specificity of algorithms in mathematical models can overestimate the impact of case-finding strategies and mislead NTPs toward harmful decisions. In order to improve model estimates and strengthen programmatic decisions, more empirical research is needed on the accuracy of WHO-recommended screening and diagnostic tools, especially clinical diagnosis and the influence of chest radiography.

## Additional file

Additional File 1: Model parameters. Parameter values for each modelled scenario. (DOCX 19366 kb)

## Abbreviations

ACF: Active case finding
DOTS: Directly observed therapy short-course
FP: False positive
ICF: Intensified case finding
NNS: Number needed to screen
NTP: National TB control programme
OPD: Outpatient department
PPV: Positive predictive value
TB: Tuberculosis
TIME: TB impact model and estimates
TP: True positive
WHO: World Health Organization
