# Supplementary material for: Investigating the impact of TB case-detection strategies and the consequences of false positive diagnosis through mathematical modelling
Source: BMC Infect Dis. 2018 Jul 21;18:340. doi: 10.1186/s12879-018-3239-x (PMC6054844; doi:10.1186/s12879-018-3239-x)
Supplement: Supplementary file 1 — Model parameters. Parameter values for each modelled scenario. (DOCX 19366 kb) [file 12879_2018_3239_MOESM1_ESM.docx]

**Investigating the impact of TB case-detection strategies and the consequences of false positive diagnosis through mathematical modelling**

Additional file 1

**Table A1.** Model care cascade parameters for baseline and intervention scenarios

|  | **Baseline** | **S1A** | **S1B** | **S2A** | **S2B** |
| --- | --- | --- | --- | --- | --- |
|  | **2017** | **2020** | **2020** | **2020** | **2020** |
| **New, smear positive screening rate** | 108 | 125 | 125 | 112 | 112 |
| **Relative screening rate, smear negative** | 0.2 | 0.2 | 0.2 | 0.2 | 0.2 |
| **Relative screening rate, previously-treated** | 1 | 1 | 1 | 1 | 1 |
| **Relative screening rate, disease-free population** | 0.003 | 0.003 | 0.003 | 0.006 | 0.006 |
| **Smear positive net sensitivity** | 0.5 | 0.499 | 0.554 | 0.5 | 0.491 |
| **Smear negative net sensitivity** | 0.209 | 0.218 | 0.209 | 0.209 | 0.278 |
| **Net specificity** | 0.949 | 0.956 | 0.948 | 0.949 | 0.999 |
| **Drug sensitive linkage to care** | 0.82 | 0.82 | 0.82 | 0.82 | 0.82 |
| **Drug sensitive treatment success** | 0.84 | 0.84 | 0.84 | 0.84 | 0.84 |
| **MDR case detection: new cases** | 0 | 0 | 0 | 0 | 0 |
| **MDR case detection: previously-treated cases** | 0.4 | 0.4 | 0.4 | 0.4 | 0.4 |
| **MDR linkage to care** | 0.4 | 0.4 | 0.4 | 0.4 | 0.4 |
| **MDR treatment success** | 0.33 | 0.33 | 0.33 | 0.33 | 0.33 |

S1A = Scenario 1, Prolonged cough & GeneXpert; S1B = Scenario 1, Any symptom & microscopy/clinical diagnosis; S2A = Scenario 2, Prolonged cough & microscopy/clinical diagnosis; S2B = Scenario 2, Prolonged cough & GeneXpert.

Screening rate for smear negative, previous-treated and disease-free population are relative to new, smear positive screening rate. Sensitivity and specificity parameters presented for the scenarios are weighted average based on coverage of the intervention and baseline parameter values.

MDR case detection should be interpreted as the coverage of drug susceptibility testing amongst new and previous-treated TB cases.

Linkage to care refers to the proportion of individuals diagnosed with TB (false and true positive cases) who are started on anti-TB treatment – this is equal to the notified population.

Treatment success is the proportion of individuals who started anti-TB treatment and have been successfully treated.

Scenario parameters that have been changed from baseline values are highlighted in green.

**Table A2.** Model natural history parameters

| **Effective contact rate** | 13.1 |
| --- | --- |
| **Proportion of cases developing smear positive TB, HIV-** | 0.4 |
| **Proportion of cases developing smear positive TB, HIV+** | 0.327 |
| **Relative infectiousness smear negative TB, HIV-** | 0.25 |
| **Relative infectiousness smear negative TB, HIV+** | 0.25 |
| **Smear conversion rate, HIV-** | 1 |
| **Smear conversion rate, HIV+** | 2.25 |
| **Self-cure rate, HIV-** | 0.2 |
| **Self-cure rate, HIV+** | 0.1 |
| **Relative fitness of MDR strains, HIV-** | 0.75 |
| **Relative fitness of MDR strains, HIV+** | 0.75 |
| **Rate of acquiring MDR, HIV-** | 1 |
| **Rate of acquiring MDR, HIV+** | 1 |
| **Relative treatment success of first-line anti-TB treatment for MDR treatment naïve, HIV-** | 0.61 |
| **Relative treatment success of first-line anti-TB treatment for MDR treatment naïve, HIV+** | 0.61 |
| **Relative treatment success of first-line anti-TB treatment for MDR previously-treated, HIV-** | 0.4 |
| **Relative treatment success of first-line anti-TB treatment for MDR previously-treated, HIV+** | 0.4 |
| **Proportion of infections developing primary TB, HIV-** | 0.115 |
| **Proportion of infections developing primary TB, HIV+** | 0.299 |
| **Reactivation rate, HIV-** | 0.12 |
| **Reactivation rate, HIV+** | 0.38 |
| **Protection provided by prior infection, HIV-** | 0.65 |
| **Protection provided by prior infection, HIV+** | 0.325 |
| **Smear positive TB mortality rate, HIV-** | 0.2 |
| **Smear positive TB mortality rate, HIV+** | 0.82 |
| **Smear negative TB mortality rate, HIV-** | 0.18 |
| **Smear negative TB mortality rate, HIV+** | 0.5 |

Infectiousness of smear negative TB is relative to smear positive TB within the same HIV stratum. Fitness of MDR strains is relative to drug sensitive strain within the same HIV stratum. Relative treatment success of first-line anti-TB treatment for MDR is relative to treatment success of first-line anti-TB treatment for drug sensitive TB within the same HIV and treatment history stratum.

**Figure A1.** Modelled projections for Scenario 1.


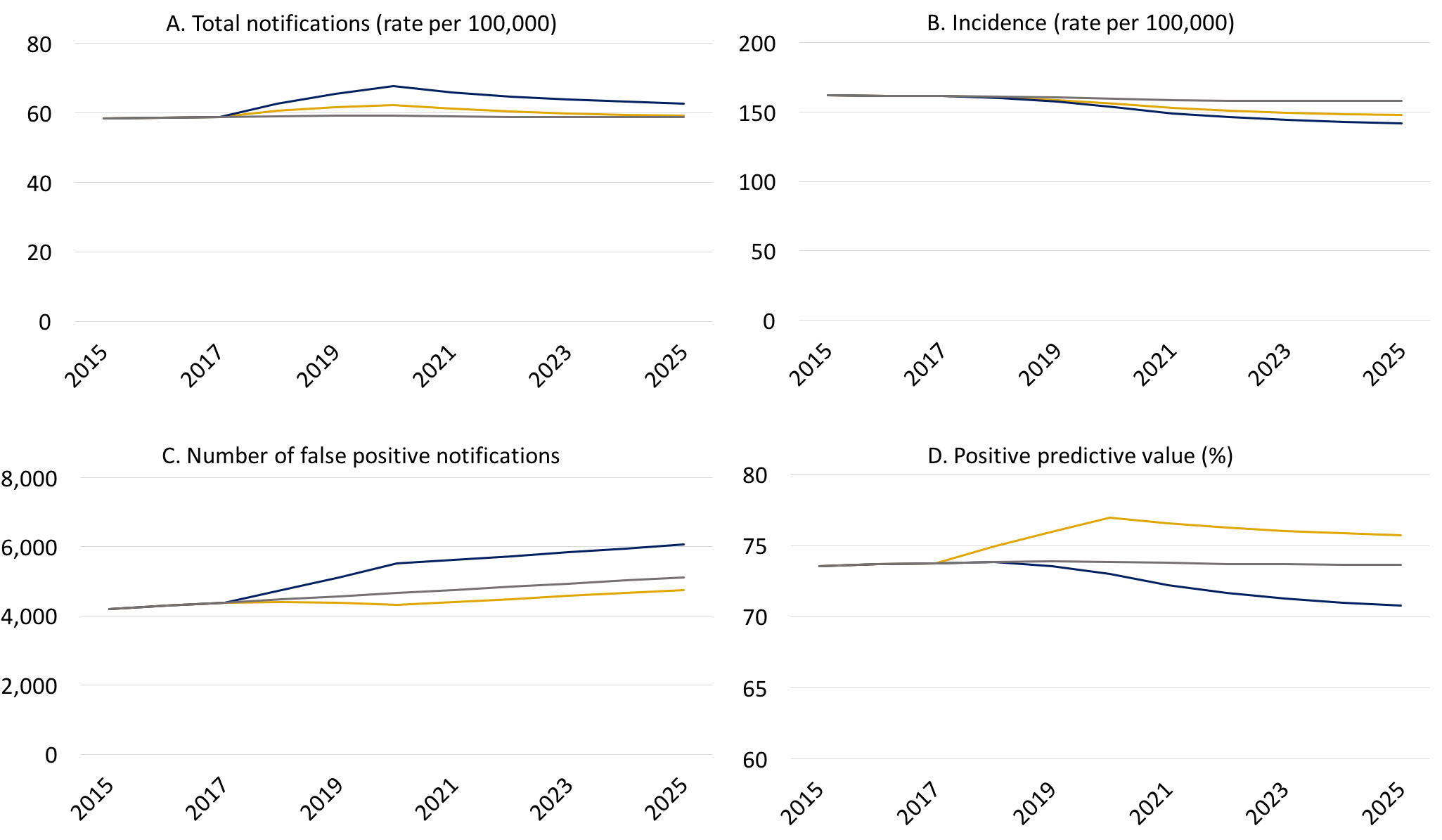


Grey = baseline; Yellow = Algorithm A (Prolonged cough & GeneXpert); Blue = Algorithm B (Any symptom & microscopy/clinical diagnosis).

**Figure A2.** Modelled projections for Scenario 2.


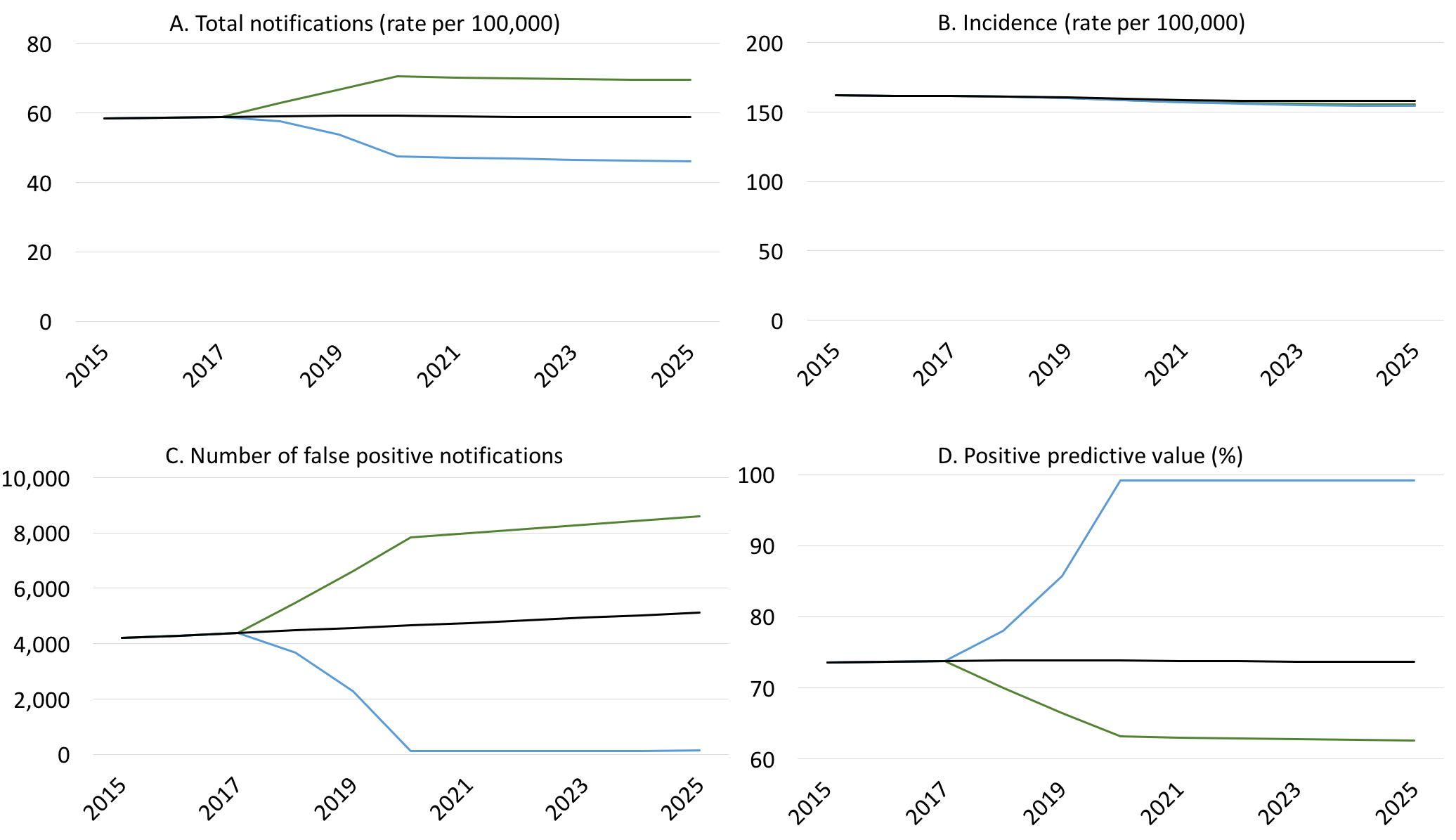


Grey = baseline; Green = ICF with microscopy/clinical diagnosis; Blue = ICF with GeneXpert
